# Design considerations for miniaturized biosensor-enabled systems for older adults: insights from participatory design

**DOI:** 10.3389/fbioe.2026.1795304

**Published:** 2026-04-01

**Authors:** Mia Quidato, Merlin Teodosia Suarez, Ryan Ebardo

**Affiliations:** 1 De La Salle University, Manila, Philippines; 2 University of Saint La Salle, College of Computing Studies, Information Technology Department, Bacolod, Philippines; 3 De La Salle Lipa, Lipa, Philippines

**Keywords:** health and wellness, miniaturized, older adults, participatory design, review, thematic framework

## Abstract

Miniaturized technologies, including small, portable, or wearable devices such as biosensors, smartwatches, and implantable devices, are transforming health and wellness by enabling real-time, personalized monitoring and interventions, offering older adults (OAs) greater engagement with digital health and support for self-management and well-being. OAs, however, often face usability challenges due to changes in vision, mobility, cognition, and digital literacy, highlighting the need for inclusive design. Participatory design (PD) engages OAs directly, ensuring technologies are meaningful, functional, and relevant to their everyday lives. This mini-review identifies how participatory design has been applied to emerging technologies for older adults and derives inclusive design considerations for the co-design of miniaturized, biosensor-enabled systems. A total of 22 studies published between 2020 and 2025 were synthesized and categorized into digital health technologies and civic platforms, human–robot interfaces (HRIs) for socially assistive/facilitative robots, voice-based interfaces, and immersive visualizations. Analysis revealed recurring considerations, including clarity, usability, engagement, and trustworthiness, informing a framework for age-inclusive, human-centered, miniaturized biosensor-enabled technologies.

## 1 Introduction

Miniaturized biosensor technologies are enabling the development of mobile health interfaces and integrated systems that support personalized diagnosis and interventions (Tsebesebe et al., 2025). Miniaturization reduces the physical size of technological components, wearables, and biosensor-integrated interfaces while maintaining or enhancing functionality (Goel and Kumar 2025; Tsebesebe et al., 2025). Miniaturization introduces new usability accompanied by sociotechnical challenges for OAs (Correia et al., 2021; Duarte et al., 2025; Liu et al., 2022), who may experience changes in vision, motor control, cognitive and emotional capacities, and digital literacy. Participatory design (PD) is a collaborative, user-centered approach that ensures technology meets users’ needs and perspectives (Sani et al., 2021; Desai et al., 2023; Fraune et al., 2022). Engaging OAs in PD promotes usability, acceptance, and need-based technology solutions (Duque et al., 2019). Human-centered design practices and co-creative principles (Muller, 2003) reflecting OAs’ experiences, realities, health goals, and sociotechnical context are often overlooked in biosensor-driven systems. Biosensor-enabled wearable devices are emerging as a practical approach to healthcare and monitoring that is central to personalized care (Alpert et al., 2024; Sharma et al., 2021; Mukherjee et al., 2025).

A targeted Scopus search (2020–2025) identified 22 open-access, English-language studies on older adults and participatory or co-design approaches in health and wellness technologies. Studies unrelated to interface design, usability, or technology were excluded. Included were digital health technologies and civic platforms (10), human–robot interfaces (HRIs) for socially assistive/facilitative robots (7), voice-based interfaces (3), and immersive visualization (2). All studies used participatory methods that engaged OAs in the design, development, adaptation, or evaluation of these technologies. This rapid, mini-review examines how participatory design captures older adults’ perspectives to guide the interface design of miniaturized biosensor-enabled systems. This type of review is appropriate for probing the current literature and charting the future theoretical and methodological directions for areas that need a multidisciplinary lens, such as technology, health, and gerontology (Sabiston et al., 2022). Older adults as active contributors in participatory design enhance the usability and adoption of health technologies. This article presents a thematic framework and key interface design considerations to guide co-design strategies, emphasizing that technologies for aging populations should be developed with older adults, not simply “for” them. This work aims to answer the following research questions:

RQ1: How can older adults’ perspectives guide the co-design of miniaturized biosensor systems for health and wellness?
RQ2: How can a thematic framework guide the co-design of miniaturized biosensor systems with older adults?

## 2 Inclusive participatory design trends and co-designed technologies for older adults

The studies span diverse technological solutions, reflecting the integration of interactive and assistive systems in health and wellness. They emphasize participatory and co-design approaches that enhance usability, accessibility, and meaningful engagement among older adults. Table 1 summarizes the co-designed technology areas explored in these PD studies.

**TABLE 1:** Summary of reviewed studies incorporating participatory design.

Participatory design studies with older adults		
Study No.	Author (year)	Co-design technologies and tools
Digital health technologies (DHTs) and civic platforms		
S1	Moral et al. (2023)	Integrated home care system
S2	Aslanoğlu et al. (2024)	Public-participation GIS
S3	Peng et al. (2024)	Chatbots
S4	Zhang et al. (2024)	Tele-education
S5	Guadagno et al. (2025)	Digital solutions
S6	Kokorelias et al. (2025)	Virtual care techs
S7	Rosa et al. (2025)	Health apps
S8	Theisz et al. (2025)	Digital health platforms
S9	Tunc et al. (2025)	Digital and low-tech tools
S10	Zhao et al. (2025)	Information and communications technology (ICT) tools
HRIs for socially assistive/facilitative robots		
S11	Schwaninger et al. (2021)	Assistive robots
S12	Fraune et al. (2022)	Socially facilitative robots
S13	Gasteiger et al. (2022)	Assistive health robots
S14	Olivier et al. (2023)	Social mobile robotic solution
S15	Mois et al. (2023)	Socially assistive robots
S16	Ostrowski et al. (2024)	HRIs
S17	Trinh et al. (2024)	Socially assistive robots
Voice-based interfaces (VUIs)		
S18	Desai et al. (2023)	VUIs
S19	Hu et al. (2024)	VUIs
S20	So et al. (2024)	VFAIs
Immersive visualization (augmented reality (AR) and virtual reality (VR))		
S21	Muñoz et al. (2022)	VR exergames
S22	Zapata-Restrepo et al. (2025)	AR/VR tech

### 2.1 DHT and civic platforms

Most PD studies focused on digital solutions and civic platforms supporting older adults’ health, service access, and social engagement. These tools promote independence, although adoption can be limited by perceived benefits, literacy, and access (Chadwick et al., 2024; Bertolazzi et al., 2024). Participatory initiatives have co-designed tele-education programs, home care systems, and chatbots to support older adults (Zhang et al., 2024; Moral et al., 2023; Peng et al., 2024). Challenges in health education, misinformation, and technology engagement often stem from usability issues and interpersonal dynamics, emphasizing the need for PD addressing age-related declines, technology access, digital literacy, transparency, privacy, conflict mediation, and autonomy (Peng et al., 2024; Zhang et al., 2024). Moral et al. (2023) co-designed a frailty home-monitoring system with sensors for gait, lower-limb strength, and weight. The system was paired with a mobile app that guided OAs through tests and revealed user and environmental errors. The study demonstrated how their input can inform biosensor-based health technologies. Another study used experience-based co-design to engage OAs living with HIV as partners, enabling them to share perspectives on virtual technology, identify contextual needs, and support autonomy (Kokorelias et al., 2025).

Other applications include low-contact newspaper co-design, which cultivated empathy, reduced bias, and addressed loneliness (Guadagno et al., 2025), and digital and civic co-creation, which leveraged older adults’ input to enhance social engagement, accessibility, and age-friendly urban environments. Examples include community gardening programs in Japan (Zhao et al., 2025), the *S.O.S. Idoso* health app in Brazil (Rosa et al., 2025), and age-friendly mapping tools in European cities using public-participation GIS (Aslanoğlu et al., 2024), supporting movement, social interaction, and access to services. Low-contact co-design using digital and low-tech storytelling, scenario-building, and remote participation addressed loneliness, mobility limitations, and geographic constraints (Tunc et al., 2025). Brain health literacy platforms for Alzheimer’s disease emphasized inclusive, culturally relevant, community-centered design, reducing research barriers and promoting engagement (Theisz et al., 2025).

### 2.2 HRIs for socially assistive/facilitative robots

The reviewed studies examined socially assistive and facilitative robots, emphasizing support for daily activities, companionship, and cognitive or physical aid, while informing the co-design of usable, meaningful robots for older adults. The study examined OAs’ perceptions, emotional responses, and preferred features of the *Misty* robot for home care companionship (Trinh et al., 2024). Mois et al. (2023) used questionnaires, interviews, and discussions to co-design conceptual robots for daily living, cognition, medication reminders, and emotional wellbeing (Mois et al., 2023). The *TIAGo* Iron robot was co-designed with nursing home residents and staff as a social mobile robotic solution, using cultural probes and interviews to inform personas and scenarios (Olivier et al., 2023). A year-long PD study examined preferences for robot-supported tasks and showed strong acceptance of practical support (e.g., reminders, scheduling) alongside hesitation due to privacy concerns (Ostrowski et al., 2024). Cross-cultural co-design of socially facilitative robots could identify behaviors and adjust interactions and appearance to fit daily routines and social needs (Fraune et al., 2022). A multi-phase international study developed *Bomy,* a home-based assistive health robot supporting medication, cognition, mood, and daily activities (Gasteiger et al., 2022). Through elicitation cards, PD addresses trust, independence, and well-being, grounding OAs’ personal contexts (Schwaninger et al., 2021).

### 2.3 Voice-based interfaces

Following HRIs, voice-based interfaces are emerging technologies that enable OAs to interact with devices using spoken commands, enhancing accessibility, engagement, and daily functioning. A systematic literature review highlights voice user interfaces (VUIs) that support older adults’ daily activities, health monitoring, and reminiscence therapy while emphasizing inclusive design (Deshmukh and Chalmeta, 2024). Smart speakers and voice agents were co-designed to support health education, exercise, and well-being for OAs, including those with motor or visual challenges (Hu et al., 2024). Programs like *Workout Pal* refined conversational flows through participatory approaches, emphasizing voice design, individualization, and independence (Desai et al., 2023), while voice-first ambient interfaces (VFAIs) supported real-time health monitoring, autonomy, and ethical considerations such as privacy and user agency (So et al., 2024). By understanding OAs’ sensory, emotional, and aesthetic preferences, trust, sociability, and individualization needs (Hu et al., 2024; Desai et al., 2023; So et al., 2024), participatory design enables voice-controlled systems to inform biosensor-enabled technologies that promote health, well-being, and daily life.

### 2.4 Immersive visualization (AR/VR)

Augmented and virtual reality offer immersive systems that support health, wellness, and engagement in OAs. VR exergames enhance cognitive, physical, and social health (Song et al., 2025), while AR/VR applications in healthcare and education improve therapy, training, and wellness management, although customization gaps remain (Iqbal et al., 2024; Al-Ansi et al., 2023). PD studies using workshops and prototyping created *EngAGE4Change*, which co-designed outdoor spaces to promote movement and reduce isolation, and *Seas the Day*, a VR exergame supporting exercise, cognition, and social engagement in people with dementia or mild cognitive impairment (Zapata-Restrepo et al., 2025; Muñoz et al., 2022). These findings show how older adults’ input can guide the design of interactive and biosensor-enabled technologies to support health, wellness, and daily activities while accommodating sensory limitations (Muñoz et al., 2022).

### 2.5 Inclusive participatory strategies for technology design

Table 2 summarizes participatory strategies, exploration and needs assessment, ideation and brainstorming, co-design and co-creation, experience-based co-design (EBCD), and iteration and evaluation. Direct collaboration ensures that technologies, including biosensor-enabled devices, are usable, engaging, tailored to everyday realities, and support adoption and sustained use among OAs.

**TABLE 2:** Inclusive tech design: strategies, stages, purpose, activities, and tools.

Strategy (stage)	Study (author)	Purpose	Participatory activity	Tool
Explorations and needs assessment (early stage)	S1, S3, S4, S5, S8, S9, S10, S13, S14, S16, S17, S18, S19, S20, S21, and S22	Build empathy and explore participants’ needs and experiences	Interviews, focus groups, observations, diaries, logs, sensors, and toolkits	Checklists, recording and sketching tools, notes, cameras, surveys, and digital/visual aids
Ideation and brainstorming (early stage)	S7, S8, S10, S11, S12, S15, S17, S19, S20, S21, and S22	Ideation, concept refinement, and sociotechnical exploration	Literature review and participatory methods (prototyping, interviews, surveys, and workshops) for ideation and interface analysis	Paper-based, sketching, digital, and mind-mapping aids
Co-design and co-creation (early to mid-stages)	S1 − S22	Collaboration for products/services, initiatives, and interventions	Interviews, focus groups, workshops, and design refinement	Prototyping, recording, visualization, role-playing, and online discussion tools
EBCD (early to mid-stages)	S6	Engage participants to ensure usability, relevance, and acceptance	Participatory workshops with multilingual support	Multilingual materials, low-fidelity prototypes, sketches, and inclusive tools
Iteration and evaluation (mid to later stages)	S1, S2, S3, S4, S5, S7, S8, S12, S13, S14, S15, S16, S21, and S22	Refine iteratively through prototyping, testing, and feedback	Iterative design, testing, and refinement	Prototypes, testing tools, interviews and surveys, recordings, logs, dashboards, and reflection notes

## 3 Common user-centered design insights from older adults

Older adults’ perspectives in participatory and co-design processes can provide a foundation for developing miniaturized biosensor systems for health and wellness by emphasizing clarity, usability, engagement, and trust, ultimately supporting inclusive, accepted, and sustainable technologies with meaningful health outcomes.

### 3.1 Clarity

Clarity relies on simplified, jargon-free language and contextually relevant, multimodal instructions. Larger fonts, optimized icons, colors, visual and verbal cues, and structured step-by-step guidance reduce cognitive load, build confidence, and improve comprehension. Feedback-informed adjustments to icon size, color, symbols, and accessibility features (e.g., screen reader compatibility) further enhance usability. Tailoring content and multimodal delivery to health needs and local contexts supports effective use, adoption, flexibility, and sustained engagement across diverse applications, such as tele-education and health management for older adults (Zhao et al., 2025; Theisz et al., 2025; Guadagno et al., 2025; Moral et al., 2023; Aslanoğlu et al., 2024; Kokorelias et al., 2025; Zhang et al., 2024).

### 3.2 Usability

Usability emphasizes intuitive navigation, minimal task complexity, and adaptable multimodal inputs (voice, visual, and tactile) that accommodate age-related sensory, motor, and cognitive changes. Participatory design studies reveal how real-world challenges, including touch sensitivity, dragging difficulties, color contrast issues, and preferences for familiar voice accents or read-aloud functions, inform interface design and support features. Clear feedback, personalization, trusted information, and customizable options, such as adjustable speech speed and contrast, enhance learnability, practicality, simplicity, and long-term engagement across diverse digital and robotic tools (Aslanoğlu et al., 2024; Moral et al., 2023; Mois et al., 2023; Olivier et al., 2023; Gasteiger et al., 2022; Muñoz et al., 2022; Theisz et al., 2025; Hu et al., 2024).

### 3.3 Engagement

Engagement is strengthened through personalization, meaningful feedback, immersive and aesthetically appealing interactions, and socially supportive features (Kokorelias et al., 2025; Muñoz et al., 2022). Participatory design experiences, including co-design and tailored content, contribute to enjoyment, ownership, and continued use (Hu et al., 2024; Desai et al., 2023; Moral et al., 2023). These engagement mechanisms are most effective for health-related functions such as medical adherence, memory support, and physical activity, whereas repetitive or socially complex tasks may require additional motivational strategies to sustain participation, belonging, and learning (Zapata-Restrepo et al., 2025; Tunc et al., 2025; Mois et al., 2023; Ostrowski et al., 2024; Trinh et al., 2024; Gasteiger et al., 2022).

### 3.4 Trustworthiness

Trustworthiness supports sustained acceptance and depends on transparency, user control, privacy protection, and cultural relevance (Schwaninger et al., 2021). Participatory design addresses ethical concerns, supports informed decision-making, and strengthens user agency (Zhang et al., 2024; Ostrowski et al., 2024). Familiarity, trusted sources, and interface preferences (e.g., voice systems or accents) enhance confidence, whereas technological anxiety, security concerns, practical difficulties, or social isolation can reduce understanding, reliance, and trust (So et al., 2024; Hu et al., 2024; Peng et al., 2024; Rosa et al., 2025; Fraune et al., 2022).

## 4 User-centered thematic framework for inclusive miniaturized biosensor technology

The use of wearable biosensor technologies for health monitoring has grown rapidly, especially through miniaturized systems in everyday devices like smartwatches. These technologies enable continuous tracking of physiological and behavioral data, supporting preventive care, self-management, and aging in place. Yet most commercial systems are developed in laboratories or industry, where design decisions prioritize technical feasibility and market goals over older adults’ lived experiences. As a result, interfaces may not fully match users’ realities, capabilities, routines, and values. Studies on DHTs and civic platforms, socially assistive/facilitative robots, voice-based interfaces, and AR/VR show that participatory design (PD) enhances usability for older adults. Involving users in co-design ensures interfaces are intuitive, contextually relevant, and accessible, reducing cognitive load and accommodating diverse abilities and literacy levels. Participation also supports clear instructions, adaptive interactions, personalized feedback, and socially meaningful engagement, reinforcing autonomy, trust, and sustained use.

**FIGURE 1:**
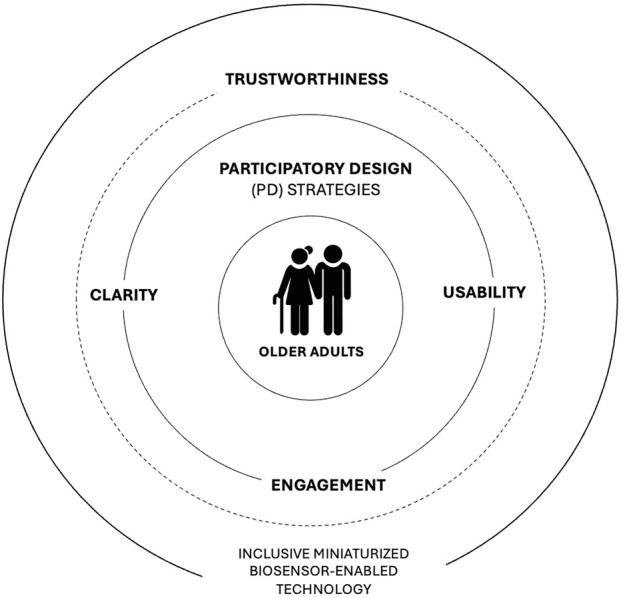
Thematic diagram for inclusive miniaturized biosensor-enabled technology.

Figure 1 positions older adults at the center, highlighting their role as active contributors rather than passive users. Their experiences, realities, preferences, and needs form the core design knowledge guiding the system. In this context, participatory design (PD) strategies create an enabling, iterative structure that supports two-way communication, reflection, and mutual learning throughout the co-design process. The next layer emphasizes clarity, usability, and engagement, showing how inclusive interfaces support adoption and sustained use of technology, enabling long-term use even in the face of challenges. The dotted-line circle represents trustworthiness, an overarching, context-dependent, and evolving construct, continuously reinforced through transparent systems, reliable feedback, and aligned behavior. The outermost layer depicts the resulting inclusive, biosensor-enabled technology, illustrating that effective outcomes emerge from participatory processes, thematic integration, and sustained trust, rather than from technical design alone.

## 5 Implications for design and future research

User-centered design insights such as clarity, usability, engagement, and trustworthiness provide a strong foundation for developing smartwatch and miniaturized biosensor technologies that support autonomy, competence, and sustained intrinsic motivation among older adults. These insights address RQ1 by showing how older adults’ perspectives guide co-design to ensure technologies are inclusive, meaningful, and used long-term. The thematic framework developed in this study addresses RQ2 by demonstrating how participatory strategies, interface principles, and trust mechanisms structure co-design processes to produce effective and widely adopted wearable technologies. Designers should integrate iterative participatory practices across all stages, from interface development to feature prioritization and testing, to maintain usability, relevance, and engagement. Future research should explore these technologies in physical activity, health monitoring, self-management, and routine formation, guided by theories such as Self-Determination Theory and Gibson’s Affordances, while also evaluating usability, integration with caregivers and programs, and ethical, privacy, and social impacts to ensure technologies remain safe, equitable, and meaningful for older adults.
