# Supplementary material for: Design considerations for miniaturized biosensor-enabled systems for older adults: insights from participatory design
Source: Front Bioeng Biotechnol. 2026 Apr 1;14:1795304. doi: 10.3389/fbioe.2026.1795304 (PMC13079303; doi:10.3389/fbioe.2026.1795304)
Supplement: Supplementary file 1 [file Supplementaryfile1.docx]

Supplementary Material

## Supplementary Table (s)

Table 1 summarizes participatory strategies, exploration and needs assessment, ideation and brainstorming, to co-design and co-creation (across all reviewed studies), an Experience-Based Co-Design, and iteration and evaluation. Direct collaboration ensures technologies, including biosensor-enabled devices, are usable, engaging, tailored to everyday realities, and support adoption, and sustained use among older adults.

**Table 1** Summary of Strategies, Stages, Purpose, Activities and Tools Used for Inclusive Tech Design

| **Common Participatory Strategies (Stages)** | **Representative Studies (Authors)** | **Purpose** | **Participatory Activities** | **Tools** |
| --- | --- | --- | --- | --- |
| Explorations and Needs Assessment (Early Stage) | (Zapata-Restrepo et al. 2025; Muñoz et al. 2022; Hu et al. 2024; So et al. 2024; Desai et al. 2023; Guadagno et al. 2025; Zhang et al. 2024; Peng et al. 2024; Theisz et al. 2025; Tunc et al. 2025; Moral et al. 2023; Trinh et al. 2024; Ostrowski et al. 2024; Zhao et al. 2025; Olivier et al. 2023; Gasteiger et al. 2022) | Build empathy, explore context, identify cases, and assess participants’ needs while capturing experiences and perceptions. | Interviews, focus groups, observations, diaries, logs, sensors, and toolkits capture older adults’ experiences to guide interventions. | Guides, checklists, recorders, sketching kits, notes, cameras, surveys, whiteboards, markers, tablets, and mind-mapping tools. |
| Ideation and Brainstorming (Early Stage) | (Zapata-Restrepo et al. 2025; Muñoz et al. 2022; So et al. 2024; Hu et al. 2024; Theisz et al. 2025; Zhao et al. 2025; Rosa et al. 2025; Trinh et al. 2024; Mois et al. 2023; Fraune et al. 2022; Schwaninger et al. 2021) | Generate and refine ideas, prioritize concepts, and discuss social and technical perspectives. | Literature review, simplified designs, interviews, surveys, discussions, demos, and workshops to generate ideas and analyze interfaces. | Whiteboards, sticky notes, markers, pens, large sheets, idea cards, sketching kits, tablets, mind-mapping tools, and interface analysis workshops. |
| Co-design and co-creation (Early to Mid-Stages) | Across representative studies | Collaborate with OAs, communities and teams to co-create products/services, plan interventions, and develop user-centered, adaptable, empathetic solutions. | Interviews, focus groups and workshops, discuss barriers and facilitators refining design principles. | Prototyping tools, devices, recordings, storyboards, personas, mock-ups, role-playing props, surveys, online conferencing, and discussion circles. |
| Experience-Base Co-Design (Early to Mid-Stages) | (Kokorelias et al. 2025) | Engage participants throughout design to grounded interventions with real-world experiences, improving relevance, usability, and acceptance. | Participatory workshops with users, supported by translation services and multilingual materials. | Translation/multilingual materials, low-fidelity prototypes, sketches, and inclusive resources for equitable participation. |
| Iteration and Evaluation (Mid to Later Stages) | (Zapata-Restrepo et al. 2025; Muñoz et al. 2022; Guadagno et al. 2025; Zhang et al. 2024; Peng et al. 2024; Theisz et al. 2025; Rosa et al. 2025; Aslanoğlu et al. 2024; Moral et al. 2023; Ostrowski et al. 2024; Mois et al. 2023; Olivier et al. 2023; Fraune et al. 2022; Gasteiger et al. 2022) | Iteratively refine designs through prototyping and testing, assess usability and effectiveness, and integrate participant feedback to enhance accessibility, engagement, and adoption. | Prototyping, testing, evaluation cycles, collecting feedback, analyzing data, updating prototypes, and adjusting processes iteratively. | Low- to high-fidelity prototypes, user testing software, interview scripts, feedback forms, SUS/UEQ-S questionnaires, recordings, logs, dashboards, reflection notes, field trials, and discussion circles. |

## Supplementary Figure (s)


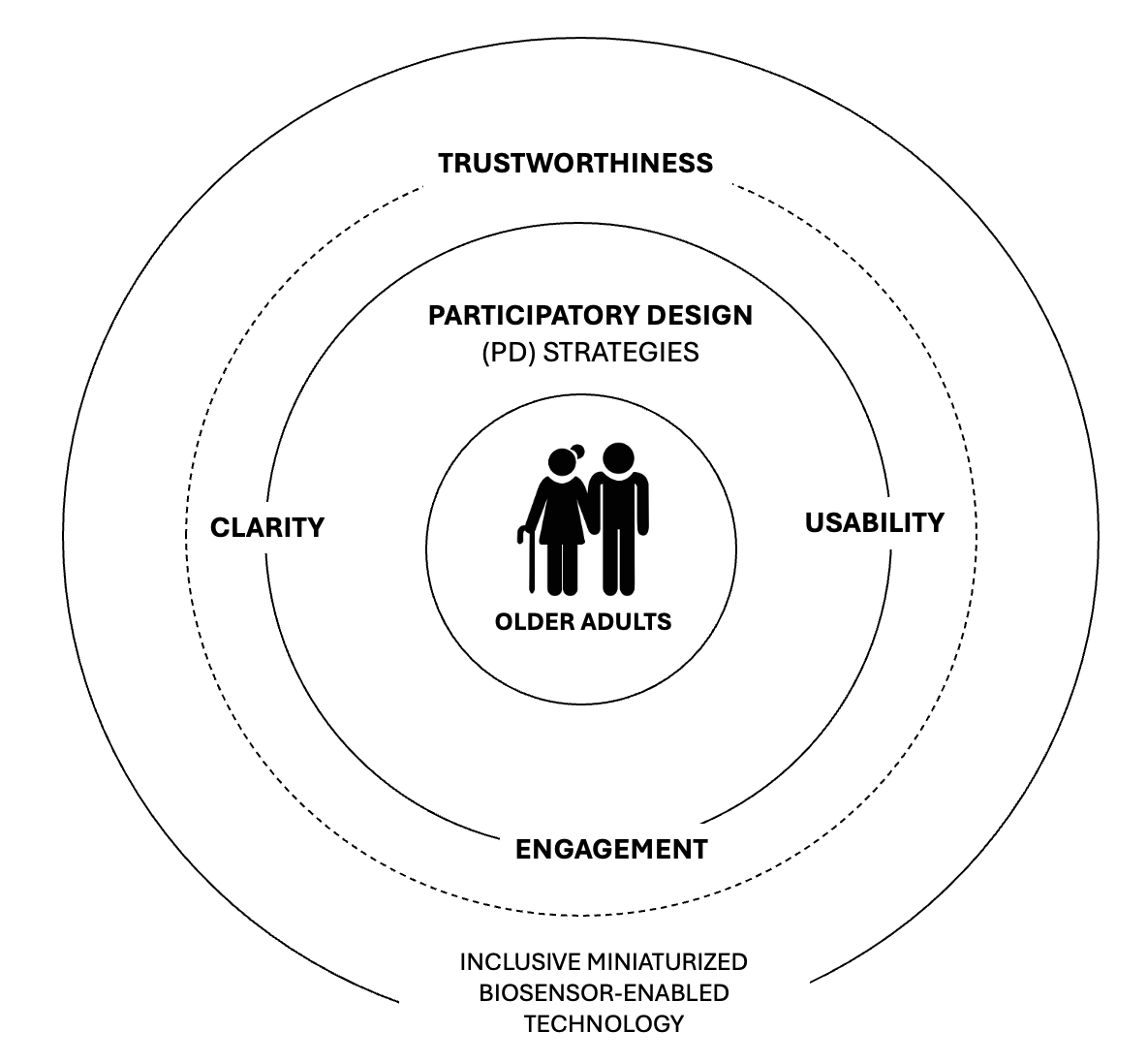


**Figure 1** Thematic Diagram for Inclusive Miniaturized Biosensor-Enabled Technology

The diagram on Figure 1 Older adults as designers at the center, emphasizing their role as active design contributors rather than passive users with their everyday realities, preferences, lived experience and needs as core design knowledge. Surrounding this, the participatory design (PD) strategies depicted as an enabling, iterative structure that sustains two-way communication, reflection, and mutual learning. The next layer, the clarity, usability, and engagement, illustrating a progression in which clear inclusive user interfaces that support adoption and sustained use. A broken circle represents trustworthiness as an overarching yet evolving construct, fragile, contextual, and continuously reinforced through transparent system, behavior and reliable feedback. The outermost layer represents the resulting inclusive, biosensor-enabled technology, signaling that effective technological outcomes emerge from participatory processes, thematic integration, and sustained trust-building rather than from technical design alone.

.
